# Screening and diagnosis of gestational diabetes mellitus in routine antenatal care: A retrospective analysis from western Kazakhstan

**DOI:** 10.1016/j.pmedr.2026.103631

**Published:** 2026-09-22

**Authors:** Aliya Makasheva, Lyudmila Yermukhanova, Khatimya Kudabayeva, Saule Tazhbenova, Aliya Zhylkybekova

**Affiliations:** a Department of Public Health and Health Care, West Kazakhstan Marat Ospanov Medical University, Aktobe, Kazakhstan; b Department of Internal Diseases № 1, West Kazakhstan Marat Ospanov Medical University, Aktobe, Kazakhstan; c Department of Pathological Physiology, West Kazakhstan Marat Ospanov Medical University, Aktobe, Kazakhstan

**Keywords:** Gestational diabetes mellitus, Antenatal care, Screening, Primary healthcare, Hyperglycemia in pregnancy

## Abstract

**Objectives:**

To assess gestational diabetes mellitus (GDM) diagnosis and screening practices in routine antenatal care in Kazakhstan.

**Methods:**

A retrospective cross-sectional study used electronic medical records of pregnant women receiving antenatal care in public outpatient clinics in Aktobe and Uralsk, Kazakhstan, from August 2024 to May 2025. Data were extracted from the DamuMed electronic medical system. A total of 445 pregnant women were included, of whom 229 had at least one GDM risk factor. Descriptive statistics and chi-square tests were used to assess screening practices and factors associated with oral glucose tolerance test (OGTT) performance at 24–28 weeks of gestation.

**Results:**

Fasting plasma glucose levels of 5.1–6.9 mmol/L were identified in 22.02% of women, while 1.57% had levels ≥7.0 mmol/L. However, GDM was documented in only 1.12%. Overall, 51.46% of women had at least one GDM risk factor. Among these women, 27.07% underwent OGTT at 24–28 weeks of gestation and 12.66% received an endocrinology consultation. OGTT uptake was significantly associated with elevated fasting plasma glucose, obesity, and a history of fetal macrosomia (*p* < 0.05).

**Conclusions:**

GDM screening and diagnosis appeared suboptimal, suggesting potential underdiagnosis and gaps in implementing national recommendations. Strengthened screening and referral pathways are needed.

## Highlights

• Hyperglycemia was common, but gestational diabetes mellitus diagnosis rates were low.
• More than half of women had at least one gestational diabetes mellitus risk factor.
• Recommended oral glucose tolerance test screening was performed in only 27.07% of high-risk women.
• Endocrinology referral was uncommon among women at increased risk.
• Results indicate underimplementation of gestational diabetes mellitus screening guidelines.

## 1 Introduction

Gestational diabetes mellitus (GDM) is one of the most common metabolic disorders during pregnancy and represents a major public health concern worldwide. According to the International Diabetes Federation, hyperglycemia in pregnancy affects approximately one in six live births globally, with GDM accounting for the majority of cases (Note, n.d.-a). The prevalence of GDM varies considerably across countries and regions due to differences in population characteristics, screening strategies, and diagnostic criteria (Albairmani et al., 2025). Reported prevalence estimates range from 6.9% in the United States (Eades et al., 2024) and 10.9% in Europe (Paulo et al., 2021) to 14.0% in Africa (Sabbagh et al., 2026) 14.8% in China (Gao et al., 2019), and 22.4% in India (Mohan et al., 2025). Studies have shown that women of South Asian origin are at higher risk of developing GDM compared with women of European ancestry (Li et al., 2022; Read et al., 2021). In Kazakhstan, nationally representative data on GDM prevalence are currently unavailable. However, estimates from local studies suggest that the prevalence ranges from 4% to 11% (Danyarova et al., 2023).

The increasing prevalence of obesity, advanced maternal age, and type 2 diabetes mellitus in Kazakhstan may contribute to a growing burden of GDM in the coming years. Women diagnosed with GDM are at increased risk of developing type 2 diabetes mellitus later in life, while their offspring are at higher risk of fetal macrosomia, birth complications, obesity, and cardiometabolic disorders (Xie et al., 2022). Early detection of hyperglycemia during pregnancy is essential for preventing adverse maternal and neonatal outcomes (Duong et al., 2025). Screening for hyperglycemia in pregnancy represents a key component of antenatal care and provides an opportunity to reduce both short-term obstetric complications and long-term cardiometabolic consequences for mothers and their children (Simmons et al., 2024; Pillay et al., 2021; Makasheva et al., 2024). In 2013, the World Health Organization adopted the recommendations of the International Association of Diabetes and Pregnancy Study Groups (IADPSG), supporting universal screening using a one-step 75-g oral glucose tolerance test (OGTT) (Scheuer et al., 2023). Alternatively, some countries use a risk factor–based screening approach, in which only women identified as being at increased risk undergo further diagnostic testing. The choice of screening strategy depends on healthcare resources, cost-effectiveness considerations, and local epidemiological characteristics. Therefore, the International Federation of Gynecology and Obstetrics (FIGO) recommends context-specific screening approaches adapted to national healthcare systems (Hod et al., 2015a).

Although IADPSG-based criteria and OGTT-based screening strategies have been adopted in many countries, their implementation in antenatal care remains challenging. Studies from different healthcare settings have reported incomplete adherence to GDM screening guidelines, low OGTT uptake, and missed opportunities for timely diagnosis among women with risk factors. These gaps may be related to several factors, including limited access to laboratory testing, insufficient integration of screening into routine antenatal care, lack of awareness among pregnant women, time constraints during clinical visits, organizational barriers, and the practical burden of the OGTT itself, which requires fasting, a prolonged clinic visit, glucose intake, and repeated blood sampling (Lachmann et al., 2020; Hinneh et al., 2022; Persson et al., 2009; Godevithana and Wijesinghe, 2024). As a result, even when national or international screening recommendations are available, actual diagnostic practices may differ substantially from guideline-based care.

Kazakhstan currently follows a risk factor–based screening strategy. According to national clinical guidelines, all pregnant women undergo fasting plasma glucose assessment in early pregnancy. Women with risk factors, including previous GDM, history of fetal macrosomia, family history of diabetes, impaired glucose tolerance, body mass index ≥30 kg/m^2^, and maternal age ≥35 years, are referred for screening with a 75-g OGTT at 24–28 weeks of gestation (Note, n.d.-b). Despite the existence of national clinical guidelines, little is known about the actual implementation of GDM screening and diagnostic practices in routine antenatal care in Kazakhstan. Furthermore, no previous studies have evaluated the discrepancy between hyperglycemia detected during routine antenatal care, officially documented GDM diagnoses, and OGTT uptake at 24–28 weeks of gestation among women at increased risk. Therefore, the aim of this study was to assess the level of GDM diagnosis and the implementation of recommended screening practices within routine antenatal care services in Kazakhstan.

## 2 Materials and methods

### 2.1 Study design

This was a retrospective cross-sectional study based on patient records obtained between August 2024 and May 2025 from public outpatient clinics located in the Aktobe and Uralsk cities in western Kazakhstan.

### 2.2 Study population and data collection

The study was conducted in public outpatient clinics in Aktobe and Uralsk, two major cities in western Kazakhstan. Of the ten public outpatient clinics invited to participate, five provided approval for data collection. Lists of pregnant women receiving antenatal care during the study period were obtained from the Pregnant Women Registry of the participating public outpatient clinics. In total, 1354 pregnant women were registered for antenatal care in these clinics during the study period. The study sample was formed in two stages. First, patient records of all eligible pregnant women were consecutively reviewed and extracted from the electronic medical system DamuMed. Eligibility criteria for the overall study sample included singleton pregnancy, gestational age ≥28 weeks, and absence of severe comorbid conditions. Women with previously diagnosed type 1 or type 2 diabetes mellitus and records with incomplete data were excluded. After applying these criteria, 909 women were excluded because they did not meet one or more eligibility criteria, and 445 patient records were included in the overall analysis.

Second, an analytical subgroup was identified within the overall study sample to evaluate diagnostic practices among women at increased risk of GDM. Women were included in this subgroup if they had at least one established GDM risk factor, including previous GDM, age ≥ 35 years, first-degree family history of diabetes, body mass index (BMI) ≥30 kg/m^2^, or history of macrosomia in previous pregnancies. Overall, 229 women met at least one of these criteria and were included in the subgroup analysis. The remaining 216 women had no identified GDM risk factors and constituted the comparison group for analyses according to GDM risk status.

The extracted data included socio-demographic characteristics, clinical characteristics, laboratory parameters, and healthcare process indicators. Socio-demographic characteristics included age, marital status, and education. Clinical characteristics included number of pregnancies and deliveries, body mass index, family history of diabetes, and history of macrosomia in previous pregnancies. Laboratory parameters included initial blood glucose level and OGTT results. Healthcare process indicators included the number of physician visits among women at high risk of GDM, particularly endocrinologist consultations, as well as the performance of GDM screening at 24–28 weeks of gestation among women with risk factors. All data were anonymized, coded, and analyzed in aggregated form to ensure participant confidentiality and prevent individual identification.

### 2.3 Statistical analysis

Data accuracy was verified through double-entry checking in Microsoft Excel prior to statistical analysis. Descriptive statistics were used to summarize the socio-demographic, clinical, laboratory, and healthcare process characteristics of the study population. Categorical variables were presented as absolute numbers and percentages. Continuous variables were presented as means and standard deviations. Descriptive analysis was performed for the overall study sample of 445 pregnant women. Women were then stratified according to GDM risk status into those without identified risk factors (*n* = 216) and those with at least one risk factor (*n* = 229), and their socio-demographic, clinical, and laboratory characteristics were compared.

To assess associations between categorical variables, Pearson's chi-square test was used. Continuous variables were compared between women with and without GDM risk factors using the independent-samples *t*-test. In the high-risk subgroup, Pearson's chi-square test was applied to evaluate factors associated with the performance of OGTT testing at 24–28 weeks of gestation. Statistical significance was defined as *p* < 0.05. All statistical analyses were performed using IBM SPSS Statistics, version 26.0 (IBM Corporation, Armonk, NY, USA).

### 2.4 Ethical approval

This study was conducted in accordance with the principles of the Declaration of Helsinki. Ethical approval was obtained from the Local Ethics Committee of West Kazakhstan Marat Ospanov Medical University, Aktobe, Kazakhstan prior to the study (Protocol No. 11 dated November 28, 2023). The requirement for informed consent was waived due to the retrospective nature of the study.

## 3 Results

A total of 445 pregnant women were included in the retrospective analysis. The mean age of participants was 30.08 ± 5.94 years. The largest age group consisted of women aged 25–29 years, accounting for 28.09%, while women aged ≥35 years represented 26.74% of the study population. Most participants were married (93.71%). Regarding educational level, the majority of women had tertiary education (45.17%) or secondary education (43.15%).

Among the overall study sample, 229 women (51.46%) had at least one risk factor for GDM, while 216 women (48.54%) had no identified risk factors. In the high-risk subgroup, the mean age was higher, at 32.86 ± 6.15 years, compared with 27.13 ± 3.96 years among women without identified risk factors. The most common age categories among women with GDM risk factors were 35–39 years (40.18%), whereas among women without identified risk factors, women aged 25–29 years predominated (41.20%). Most women in both groups were married (95.20% vs. 92.13%; *p* = 0.18). Tertiary education predominated among women without GDM risk factors (45.83%), whereas tertiary and secondary education were equally common in the high-risk group (44.54% each; *p* = 0.78). Neither marital status nor educational level differed significantly between the groups (Table 1).

**Table 1:** Socio-demographic characteristics of pregnant women receiving antenatal care in public outpatient clinics in Aktobe and Uralsk, western Kazakhstan, according to the presence of gestational diabetes mellitus risk factors, from August 2024 to May 2025.

Variables	All women (*n* = 445)		No risk factors (*n* = 216)		≥1 risk factor (*n* = 229)		*p*-value
	Frequency	%	Frequency	%	Frequency	%	
Education level							0.78
Primary	52	11.69	27	12.50	25	10.92	
Secondary	192	43.15	90	41.67	102	44.54	
Tertiary	201	45.17	99	45.83	102	44.54	
Marital status							0.18
Unmarried	28	6.29	17	7.87	11	4.80	
Married	417	93.71	199	92.13	218	95.20	
Age (years)							**<0.01**
<20 years	5	1.12	3	1.39	2	0.87	
20–24 years	86	19.33	57	26.39	29	12.66	
25–29 years	125	28.09	89	41.20	36	15.72	
30–34 years	110	24.72	67	31.02	43	18.78	
35–39 years	92	20.67	0	0	92	40.18	
≥40 years	27	6.07	0	0	27	11.79	

	**Mean value**	**Standard deviation**	**Mean value**	**Standard deviation**	**Mean value**	**Standard deviation**	
Age (years)	30.08	5.94	27.13	3.96	32.86	6.15	**<0.01**

Table 2 presents the clinical and laboratory characteristics of the study population according to the presence of GDM risk factors. Fasting plasma glucose levels of 5.1–6.9 mmol/L were identified in 22.02% of pregnant women, while 1.57% had levels ≥7.0 mmol/L, meeting the diagnostic threshold for overt diabetes in pregnancy. Overall, GDM was documented in 5 women (1.12%). A family history of diabetes was reported in 8.09% of women in the overall study sample and in 15.72% of women with GDM risk factors. Among women with previous deliveries, a history of fetal macrosomia (>4000 g) was observed in 13.93% of the overall sample and in 27.07% of women with GDM risk factors.

**Table 2:** Clinical, laboratory, and healthcare process characteristics of pregnant women receiving antenatal care in public outpatient clinics in Aktobe and Uralsk, western Kazakhstan, according to the presence of gestational diabetes mellitus risk factors, from August 2024 to May 2025.

Variables	All women (n = 445)		No risk factors (n = 216)		≥1 risk factor (n = 229)		*p*-value
	Frequency	%	Frequency	%	Frequency	%	
Fasting plasma glucose 5.1–6.9 mmol/L (yes)	98	22.02	0	0	98	42.80	–
Fasting plasma glucose ≥7.0 mmol/L (overt diabetes in pregnancy) (yes)	7	1.57	0	0	7	3.06	–
Family history of diabetes (yes)	36	8.09	0	0	36	15.72	–
History of fetal macrosomia							–
No children	162	36.40	96	44.44	66	28.82	
No	221	49.66	120	55.56	101	44.11	
Yes	62	13.93	0	0	62	27.07	
Body mass index							–
Underweight <18.5	21	4.72	19	8.80	2	0.87	
Normal weight 18.5–24.9	275	61.80	159	73.61	116	50.66	
Overweight 25.0–29.9	110	24.72	38	17.59	72	31.44	
Obesity ≥30	39	8.76	0	0	39	17.03	
Number of deliveries							**<0.01**
0	130	29.21	82	37.96	48	20.96	
1	114	25.62	66	30.56	48	20.96	
2	103	23.15	43	19.91	60	26.20	
≥3	98	22.02	25	11.57	73	31.88	
Number of risk factors							–
0	216	48.54	216	100.00	0	0	
1	151	33.93	0	0	151	65.94	
2	50	11.24	0	0	50	21.83	
3	16	3.60	0	0	16	6.99	
4	11	2.47	0	0	11	4.80	
5	1	0.22	0	0	1	0.44	
Oral glucose tolerance test at 24–28 weeks (yes)	62	13.93	0	0	62	27.07	**<0.01**
Endocrinologist consultation (yes)	38	8.54	9	4.17	29	12.66	**<0.01**

	**Mean value**	**Standard deviation**	**Mean value**	**Standard deviation**	**Mean value**	**Standard deviation**	
Body mass index, kg/m^2^	24.08	4.27	22.28	2.75	25.77	4.73	–
Initial blood glucose level, mmol/L	4.75	0.73	4.46	0.43	5.03	0.84	–
Number of pregnancies	2.98	1.65	2.51	1.41	3.41	1.75	**<0.01**
Number of deliveries	1.48	1.32	1.07	1.08	1.86	1.41	**<0.01**

– *P*-values were not calculated for variables used to define the GDM risk group.

Normal body weight (BMI 18.5–24.9 kg/m^2^) was more common among women without identified risk factors than among those with GDM risk factors (73.61% vs. 50.66%), whereas overweight was more frequent among women with risk factors (31.44% vs. 17.59%). Obesity (BMI ≥30 kg/m^2^) was observed in 17.03% of women with GDM risk factors and was absent among women without identified risk factors.

The distribution of previous deliveries differed significantly between women with and without GDM risk factors (*p* < 0.01). Women with ≥3 previous deliveries accounted for 31.88% of the high-risk group compared with 11.57% among women without risk factors. Mean BMI was 25.77 ± 4.73 kg/m^2^ among women with GDM risk factors and 22.28 ± 2.75 kg/m^2^ among those without identified risk factors. The corresponding mean initial fasting plasma glucose levels were 5.03 ± 0.84 and 4.46 ± 0.43 mmol/L, respectively. Women with GDM risk factors also had a higher mean number of pregnancies (3.41 ± 1.75 vs. 2.51 ± 1.41; *p* < 0.01) and deliveries (1.86 ± 1.41 vs. 1.07 ± 1.08; *p* < 0.01).

Among the 229 women with at least one GDM risk factor, 62 (27.07%) underwent OGTT at 24–28 weeks of gestation. No early OGTT was documented. Among 105 women with fasting plasma glucose ≥5.1 mmol/L in early pregnancy, 59 (56.19%) underwent OGTT at 24–28 weeks of gestation. Among the 62 women who underwent OGTT at 24–28 weeks, GDM was documented in 5 (8.07%), all of whom subsequently received GDM management. Endocrinologist consultation was more frequent among women with GDM risk factors than among those without risk factors (12.66% vs. 4.17%; *p* < 0.01).

Table 3 presents factors associated with OGTT at 24–28 weeks of gestation among pregnant women with at least one GDM risk factor. OGTT was found to be significantly associated with elevated initial blood glucose levels, obesity, and a history of macrosomia. Among women who underwent OGTT at 24–28 weeks of gestation, elevated glucose levels were identified in 95.16% compared with 27.54% among women who did not undergo OGTT at 24–28 weeks (*p* < 0.01). Obesity (BMI ≥30 kg/m^2^) was also significantly more common among women who underwent OGTT at 24–28 weeks (38.71%; *n* = 24 vs. 8.98%; *n* = 15; *p* < 0.01). Similarly, a history of macrosomia was significantly associated with undergoing OGTT at 24–28 weeks (60.98%; *n* = 25 vs. 30.33%; *n* = 37; *p* < 0.01).

**Table 3:** Factors associated with oral glucose tolerance testing at 24–28 weeks of gestation among pregnant women with at least one gestational diabetes mellitus risk factor receiving antenatal care in public outpatient clinics in Aktobe and Uralsk, western Kazakhstan, from August 2024 to May 2025 (n = 229).

Factor	Group	Oral glucose tolerance test not performed n (%)	Oral glucose tolerance test performed n (%)	*p*
Age	<35	77 (46.11)	33 (53.23)	0.37
	≥35	90 (53.89)	29 (46.77)	

Body mass index, kg/m^2^	<30	152 (91.02)	38 (61.29)	**<0.01**
	≥30	15 (8.98)	24 (38.71)	

Initial blood glucose level, mmol/L	<5.1	121 (72.46)	3 (4.84)	**<0.01**
	≥5.1	46 (27.54)	59 (95.16)	

Family history of diabetes	No	139 (83.23)	54 (87.10)	0.55
	Yes	28 (16.77)	8 (12.90)	

History of fetal macrosomia	No	85 (69.67)	16 (39.02)	**<0.01**
	Yes	37 (30.33)	25 (60.98)	

Endocrinologist consultation	No	149 (89.22)	51 (82.26)	0.18
	Yes	18 (10.78)	11 (17.74)	

*P*-values were calculated using Pearson's chi-square test. Statistical significance was defined as *p* < 0.05.

In contrast, no statistically significant associations were observed between OGTT and age ≥ 35 years (*p* = 0.37), family history of diabetes (*p* = 0.55), or endocrinologist consultation (*p* = 0.18).

## 4 Discussion

The interpretation of hyperglycemia detected in early pregnancy requires a clear distinction between GDM and overt diabetes in pregnancy. According to WHO criteria, fasting plasma glucose of 5.1–6.9 mmol/L is classified as GDM, whereas fasting plasma glucose ≥7.0 mmol/L is classified as diabetes in pregnancy (*Diabetes Res. Clin. Pract.*, 2014). The current national clinical protocol of Kazakhstan follows the same diagnostic thresholds for hyperglycemia detected during pregnancy. For women whose fasting plasma glucose is below the diagnostic threshold for GDM but who have one or more recognized GDM risk factors, a 75-g OGTT is recommended at 24–28 weeks of gestation (Note, n.d.-b).

In our study, fasting plasma glucose levels ranging from 5.1 to 6.9 mmol/L were identified in 22.02% of pregnant women, while 1.57% had levels ≥7.0 mmol/L. However, GDM was documented in only 1.12% of women. The marked discrepancy between the proportion of women with fasting plasma glucose levels within the diagnostic range for GDM and the proportion with a documented GDM diagnosis suggests potential underdiagnosis in routine clinical practice. This gap may indicate missed opportunities for timely diagnosis and appropriate management.

More than half of the women included in this study had at least one recognized risk factor for GDM. Nevertheless, only 27.07% of these women underwent OGTT at 24–28 weeks of gestation, despite recommendations outlined in national clinical guidelines. These findings indicate insufficient coverage of screening among women at increased risk and suggest incomplete implementation of existing screening algorithms. Our findings are consistent with a population-based retrospective cross-sectional study conducted in Sweden by Persson et al., which also reported low adherence to local guidelines for risk factor–based GDM screening (Persson et al., 2009). Despite the availability of international and national recommendations for GDM screening, their effective implementation may be hindered by limited access to laboratory diagnostics (Bhavadharini et al., 2016), shortages of healthcare personnel and organizational resources (Hinneh et al., 2022), poor tolerability of the OGTT protocol among pregnant women (Lachmann et al., 2020), as well as low awareness of GDM and its potential consequences (Utz et al., 2020). The FIGO emphasizes that the effective implementation of GDM screening programs depends not only on the existence of evidence-based clinical guidelines, but also on adequate healthcare resources, a well-trained workforce, and robust monitoring and quality assurance systems (Hod et al., 2015b).

Women who underwent OGTT were more likely to have clinically significant risk factors, including elevated blood glucose levels, obesity, and a history of macrosomia. This suggests that referral for additional testing was more commonly provided to women perceived by healthcare professionals as being at higher risk of developing GDM. Although this approach is consistent with a risk-based model of clinical decision-making, it may also lead to insufficient screening of women with less pronounced risk factors who nevertheless meet the criteria for OGTT according to national recommendations (Minschart et al., 2021).

Furthermore, the very low proportion of endocrinology consultations among women with GDM risk factors (12.66%) may reflect gaps in the multidisciplinary care of high-risk pregnant women (Utz et al., 2016). This interpretation is supported by Mukona et al., who reported that barriers to diabetes care in pregnancy included lack of trained personnel, shortage of staff, and insufficient collaboration among healthcare practitioners, while recommending multidisciplinary care involving endocrinologists, obstetricians, midwives, diabetes specialist nurses, and dietitians (Mukona et al., 2017).

These findings are consistent with international evidence indicating a gap between clinical guidelines and their implementation in routine practice. Therefore, the low uptake of OGTT at 24–28 weeks of gestation, as recommended by current national clinical guidelines, may indicate incomplete implementation of existing screening algorithms and the presence of systemic barriers contributing to the underdiagnosis of GDM within routine antenatal care (Hinneh et al., 2022).

This study has several limitations. First, the retrospective design limited the ability to determine the reasons why OGTT was not performed. Second, the study relied on routinely collected electronic medical records, which may contain incomplete or inaccurately documented information. Third, the study was conducted in selected primary healthcare facilities in two cities and may not fully represent practices in other regions of Kazakhstan. In addition, only five of the ten public outpatient clinics invited to participate provided approval for data collection, which may limit the representativeness of the findings even within the two study cities. Therefore, the results should primarily be interpreted as reflecting GDM screening practices in the participating urban public outpatient clinics.

Despite these limitations, this study provides one of the first assessments of GDM screening implementation within routine antenatal care in Kazakhstan. The findings highlight important gaps between guideline recommendations and actual clinical practice. Strengthening screening coverage, improving adherence to diagnostic algorithms, and enhancing monitoring of high-risk pregnancies may contribute to earlier detection and more effective management of GDM at the primary healthcare level.

## 5 Conclusion

A substantial discrepancy was identified between the frequency of hyperglycemia detected during pregnancy and the number of officially documented GDM diagnoses. In addition, the coverage of OGTT among women with risk factors was low. These findings indicate important gaps in the implementation of GDM screening and diagnostic practices within primary healthcare services and support the need for measures aimed at improving early detection and management of GDM in Kazakhstan.

## Ethics declarations

The authors have not obtained informed consent from participants or their legal representatives. The requirement for informed consent was waived due to the retrospective nature of the study.

This study was performed in compliance with relevant laws, regulatory frameworks and guidelines where the research took place. This study was conducted in accordance with Declaration of Helsinki. This study was approved by the Local Ethics Committee of West Kazakhstan Marat Ospanov Medical University, Aktobe, Kazakhstan (Approval No. Protocol No. 11).

## CRediT authorship contribution statement

**Aliya Makasheva:** Writing – review & editing, Writing – original draft, Methodology, Investigation, Formal analysis, Data curation. **Lyudmila Yermukhanova:** Writing – review & editing, Supervision, Methodology, Formal analysis, Conceptualization. **Khatimya Kudabayeva:** Writing – review & editing, Supervision, Resources, Methodology, Formal analysis. **Saule Tazhbenova:** Writing – review & editing, Resources, Project administration, Data curation, Conceptualization. **Aliya Zhylkybekova:** Writing – review & editing, Methodology, Formal analysis, Data curation.

## Funding

This research was supported by the Non-profit Joint Stock Company West Kazakhstan Marat Ospanov Medical University (Order No. 13/2-18-433 dated May 29, 2024).

## Declaration of competing interest

The authors declare that they have no known competing financial interests or personal relationships that could have appeared to influence the work reported in this paper.

## Data Availability

Data will be made available on request.

## References

[bb0005] Albairmani R.A., Basheer B.M., Macky M.M., Al Syouti T., AlZubaidy H., Elfaki E., Kidwai A., Basheer Y.M., Ahmed F., Salaheldin M. (2025). Management of diabetes in pregnancy: a review of clinical guidelines and practices. Cureus.

[bb0010] Bhavadharini B., Uma R., Saravanan P., Mohan V. (2016). Screening and diagnosis of gestational diabetes mellitus – relevance to low and middle income countries. Clin Diabetes Endocrinol..

[bb0015] Danyarova L., Kuanyshbekova R., Svyatova G., Berezina G., Sultanova B. (2023). PS-P11-8: gestational diabetes mellitus as a predictor of type 2 diabetes mellitus: cardiovascular risk factors and biological markers in Kazakh women. J. Hypertens..

[bb0020] (2014). Diagnostic criteria and classification of hyperglycaemia first detected in pregnancy: a World Health Organization Guideline. Diabetes Res. Clin. Pract..

[bb0025] Duong T.L., Shahunja K.M., Le M., McIntyre D.H., Ward J., Mamun A.A. (2025). Gestational diabetes mellitus and its impact on maternal and neonatal outcomes in Indigenous populations: a systematic review and meta-analysis. Diabetes Res. Clin. Pract..

[bb0030] Eades C.E., Burrows K.A., Andreeva R., Stansfield D.R., Evans J.M.M. (2024). Prevalence of gestational diabetes in the United States and Canada: a systematic review and meta-analysis. BMC Pregnancy Childbirth.

[bb0035] Gao C., Sun X., Lu L., Liu F., Yuan J. (2019). Prevalence of gestational diabetes mellitus in mainland China: a systematic review and meta-analysis. J. Diabetes Investig..

[bb0040] Godevithana J., Wijesinghe C.J. (2024). Barriers and facilitators for universal gestational diabetes mellitus screening in a low resource setting: a cross-sectional study in Sri Lanka. Sci. Rep..

[bb0045] Hinneh T., Jahn A., Agbozo F. (2022). Barriers to screening, diagnosis and management of hyperglycaemia in pregnancy in Africa: a systematic review. Int. Health.

[bb0050] Hod M., Kapur A., Sacks D.A., Hadar E., Agarwal M., Di Renzo G.C., Roura L.C., McIntyre H.D., Morris J.L., Divakar H. (2015). The International Federation of Gynecology and Obstetrics (FIGO) Initiative on gestational diabetes mellitus: a pragmatic guide for diagnosis, management, and care. Int. J. Gynecol. Obstet..

[bb0055] Hod M., Kapur A., Sacks D.A., Hadar E., Agarwal M., Renzo G.C.D., Roura L.C., McIntyre H.D., Morris J.L., Divakar H. (2015). The International Federation of Gynecology and Obstetrics (FIGO) Initiative on gestational diabetes mellitus: a pragmatic guide for diagnosis, management, and care. Int. J. Gynecol. Obstet..

[bb0060] Lachmann E.H., Fox R.A., Dennison R.A., Usher-Smith J.A., Meek C.L., Aiken C.E. (2020). Barriers to completing oral glucose tolerance testing in women at risk of gestational diabetes. Diabet. Med..

[bb0065] Li L.-J., Huang L., Tobias D.K., Zhang C. (2022). Gestational diabetes mellitus among Asians – a systematic review from a population health perspective. Front. Endocrinol..

[bb0070] Makasheva A., Yermukhanova L., Kudabayeva K., Tazhbenova S., Nogayeva M., Tautanova A., Zhylkybekova A. (2024). Primary care for gestational diabetes: a bibliometric analysis of publications from 1991 to 2024. Int. J. Environ. Res. Public Health.

[bb0075] Minschart C., Beunen K., Benhalima K. (2021). An update on screening strategies for gestational diabetes mellitus: a narrative review. Diabetes Metab. Syndr. Obes..

[bb0080] Mohan V., Deepa M., Tandon N., Pradeepa R., Subashini R., Janeline L., Lakshmy R., Bajaj S., Dash K., Adhikari P., John M., Ram U., Sagili H., Sriram U., Chandhiok N., Das A.K., Unnikrishnan R., Bhatnagar S., Joshi S., Kaur T., Dhaliwal R.S., Anjana R.M. (2025). Prevalence of gestational diabetes mellitus in India: the ICMR-INDIAB national study (ICMR-INDIAB-24). Indian J. Med. Res..

[bb0085] Mukona D.M., Munjanja S.P., Zvinavashe M., Stray-Pederson B. (2017). Barriers and facilitators of adherence to anti-diabetic therapy in pregnant women with diabetes: health care workers' perspectives. J. Diabetes Mellitus.

[bb0090] International Diabetes Federation. IDF Diabetes Atlas, 11th edn. Brussels, Belgium: International Diabetes Federation, 2025, Diabetes Atlas (n.d.). https://diabetesatlas.org/ (accessed May 25, 2026).

[bb0095] Ministry of Healthcare of the Republic of Kazakhstan., Clinical Protocol No. 214 “Diabetes Mellitus During Pregnancy, Labor, and the Postpartum Period” (19 September 2024), (n.d.). https://l1nq.com/1vm8uik (accessed September 28, 2025).

[bb0100] Paulo M.S., Abdo N.M., Bettencourt-Silva R., Al-Rifai R.H. (2021). Gestational diabetes mellitus in Europe: a systematic review and meta-analysis of prevalence studies. Front. Endocrinol..

[bb0105] Persson M., Winkvist A., Mogren I. (2009). Surprisingly low compliance to local guidelines for risk factor based screening for gestational diabetes mellitus - a population-based study. BMC Pregnancy Childbirth.

[bb0110] Pillay J., Donovan L., Guitard S., Zakher B., Gates M., Gates A., Vandermeer B., Bougatsos C., Chou R., Hartling L. (2021). Screening for gestational diabetes: updated evidence report and systematic review for the US Preventive Services Task Force. JAMA.

[bb0115] Read S.H., Rosella L.C., Berger H., Feig D.S., Fleming K., Ray J.G., Shah B.R., Lipscombe L.L. (2021). BMI and risk of gestational diabetes among women of South Asian and Chinese ethnicity: a population-based study. Diabetologia.

[bb0120] Sabbagh I.T., Seedat F., Hirose A., Wade A.N. (2026). Prevalence of gestational diabetes mellitus in Sub-Saharan Africa: a systematic review and meta-analysis. AJOG Glob. Rep..

[bb0125] Scheuer C.M., Jensen D.M., McIntyre H.D., Ringholm L., Mathiesen E.R., Nielsen C.P.K., Nolsöe R.L.M., Milbak J., Hillig T., Damm P., Overgaard M., Clausen T.D. (2023). Applying WHO2013 diagnostic criteria for gestational diabetes mellitus reveals currently untreated women at increased risk. Acta Diabetol..

[bb0130] Simmons D., Gupta Y., Hernandez T.L., Levitt N., van Poppel M., Yang X., Zarowsky C., Backman H., Feghali M., Nielsen K.K. (2024). Call to action for a life course approach. Lancet.

[bb0135] Utz B., Assarag B., Essolbi A., Barkat A., Benkaddour Y.A., De Brouwere V. (2016). Diagnosis a posteriori? Assessing gestational diabetes screening and management in Morocco. Glob. Health Action.

[bb0140] Utz B., Assarag B., Lekhal T., Van Damme W., De Brouwere V. (2020). Implementation of a new program of gestational diabetes screening and management in Morocco: a qualitative exploration of health workers' perceptions. BMC Pregnancy Childbirth.

[bb0145] Xie W., Wang Y., Xiao S., Qiu L., Yu Y., Zhang Z. (2022). Association of gestational diabetes mellitus with overall and type specific cardiovascular and cerebrovascular diseases: systematic review and meta-analysis. BMJ.

